# Genetic and Historical Colonization Analyses of an Endemic Savanna Tree, *Qualea grandiflora*, Reveal Ancient Connections Between Amazonian Savannas and Cerrado Core

**DOI:** 10.3389/fpls.2018.00981

**Published:** 2018-07-17

**Authors:** Renata Santiago de Oliveira Buzatti, Thais R. Pfeilsticker, Rafael Félix de Magalhães, Marcelo L. Bueno, José P. Lemos-Filho, Maria B. Lovato

**Affiliations:** ^1^Departamento de Biologia Geral, Instituto de Ciências Biológicas, Universidade Federal de Minas Gerais, Belo Horizonte, Brazil; ^2^Programa de Pós-Graduação em Zoologia, Instituto de Ciências Biológicas, Universidade Federal de Minas Gerais, Belo Horizonte, Brazil; ^3^Departamento de Biologia Vegetal, Instituto de Ciências Biológicas, Universidade Federal de Viçosa, Viçosa, Brazil; ^4^Departamento de Botânica, Instituto de Ciências Biológicas, Universidade Federal de Minas Gerais, Belo Horizonte, Brazil

**Keywords:** Cerrado, Amazonian savanna, Vochysiaceae, Pleistocene climatic oscillation, phylogeography, colonization route, relaxed random walk, historical connection

## Abstract

The evolutionary processes underlying the high diversity and endemism in the Cerrado, the most extensive Neotropical savanna, remain unclear, including the factors promoting the presence and evolution of savanna enclaves in the Amazon forest. In this study, we investigated the effects of past climate changes on genetic diversity, dynamics of species range and the historical connections between the savanna enclaves and Cerrado core for *Qualea grandiflora*, a tree species widely distributed in the biome. Totally, 40 populations distributed in the Cerrado core and Amazon savannas were analyzed using chloroplast and nuclear DNA sequences. We used phylogeographic, coalescent and ecological niche modeling approaches. Genetic data revealed a phylogeographic structure shaped by Pleistocene climatic oscillations. An eastern-western split in the Cerrado core was observed. The central portion of the Cerrado core harbored most of the sampled diversity for cpDNA. Ecological niche models predicted the presence of a large historical refuge in this region and multiple small refuges in peripheral areas. Relaxed Random Walk (RRW) models indicated the ancestral population in the north-western border of the central portion of the Cerrado core and cyclical dynamics of colonization related to Pleistocene climatic oscillations. Central and western ancient connections between Cerrado core and Amazonian savannas were observed. No evidence of connections among the Amazonian savannas was detected. Our study highlights the importance of Pleistocene climatic oscillations for structuring the genetic diversity of *Q. grandiflora* and complex evolutionary history of ecotonal areas in the Cerrado. Our results do not support the recent replacement of a large area in the Amazon forest by savanna vegetation. The Amazonian savannas appear to be fragmented and isolated from each other, evolving independently a long ago.

## Introduction

Evolutionary processes outlining the distribution of biodiversity in South American biomes are still poorly understood, especially in the Cerrado, the most extensive Neotropical savanna, extending from 3°N to 24°S and from sea level to 1,800 m and occupying approximately 2,000,000 km^2^ (Ratter et al., 1997; Pennington et al., 2006). Its large core area coincides with the Central Brazilian Shield, with patches of characteristic vegetation occurring in north-eastern and southern Brazil as well and in disjoint areas (enclaves) in the Amazonian (Amazonian savanna) (Eiten, 1972). The Cerrado is characterized by dense grasslands, usually with shrubs and small trees growing on acidic, well-drained soil, poor in nutrients and rich in aluminum (Ratter et al., 1997). Its climate is characterized by average precipitation ranging from 800 to 2,000 mm, strong dry season, extending from April to September and average annual temperature between 18°C and 28°C (Dias, 1992).

The limited palynological data indicate that the Brazilian Cerrado became established as a large-scale vegetation during the Neogene Period (25–2 Ma; van der Hammen, 1983). During this Period, between 4 and 8 Ma, the most of biome diversification also occurred (Jacobs et al., 1999; Pennington et al., 2006; Simon et al., 2009). In the Quaternary Period, climatic oscillations altered landscape composition in South America. Palynological evidence indicates that during the glacial periods, with cooler and drier climate, savannas expanded in the east toward the Atlantic Ocean, and in the north toward the equator, while their southern portion was replaced by subtropical grasslands (Behling and Lichte, 1997; Behling, 1998). Another evidence supporting the larger extension of the Cerrado during the Quaternary Period is the presence of savanna patches within the Amazon forest (Behling, 1998; Ratter et al., 2003).

Despite recent efforts to understand past distributional patterns of the South American biota, there are numerous controversies, especially regarding the existence of glacial refuges of tropical forests in the Amazonia (Colinvaux et al., 2000; Bush and de Oliveira, 2006; Wang et al., 2017). At the Cerrado core, zones less susceptible to climatic variation have been identified, which coincide with regions harboring high species richness, endemism and genetic diversity, making them potential Quaternary refuges (Werneck et al., 2012; Bueno et al., 2017; Buzatti et al., 2017; Souza et al., 2017). Phylogeographic studies of tree species have revealed high genetic diversity in central portion of the Cerrado and low diversity in southern Cerrado and provided evidence indicating that central populations might have been sources for recent colonization of the southern Cerrado (Collevatti et al., 2003, 2009; Ramos et al., 2007; Novaes et al., 2010, 2013; Buzatti et al., 2017; Souza et al., 2017). However, thus far, just one phylogeographic plant study has ever investigated the Amazonian savannas, which revealed historical connections between south-western Amazon forest and the Cerrado core (Buzatti et al., 2017).

Several hypotheses have been proposed to explain the existence of savanna patches in the Amazon rainforest. Initially, based only on biogeographic patterns, Haffer (1969) suggested that during the dry and cool phases of the Quaternary, the Amazon forest was fragmented into small humid refuges and the fragments were separated by savannas or other dry formations. Nevertheless, palynological evidence from the Last Glacial Maximum (LGM) indicate that slight change occurred in the floristic composition of the Amazon forest and these findings have been used to refute Haffer's (1969) Pleistocene refuge hypothesis (Colinvaux, 1987; Colinvaux et al., 1996, 2000; Bush and de Oliveira, 2006; Cheng et al., 2013). However, these findings alone may not be sufficient to rule out the refuge hypothesis, since they represent single sites and only a short period of time (62 ka; Garzón-Orduña et al., 2014). According to Hooghiemstra and van der Hammen (1998), seasonal variation in precipitation in accordance to precessional cycles of orbital forcing, temperature oscillations during the Quaternary and concave shape of the Andes are the most important factors contributing to the dynamics of Amazonian vegetation, including the Amazonian savannas.

Regardless of the controversies over the extent of savanna expansion during the Late Pleistocene, the question about floristic connections between the Cerrado core and savanna enclaves in the Amazon forest remains. Three major connections between the northern and southern savannas have been proposed (Haffer, 1967, 1974; Webb, 1991): (1) Andean, linking the southern savannas with the Lhanos and the state of Roraima (northernmost part of Brazil) through the Andes; (2) Central Amazonian, connecting the southern region of savannas with savanna patches located north of the Amazonian; (3) Costal, linking the southern and northern regions through savanna patches present near the Atlantic coast. The existence of these connections is controversial, since only a few studies have found evidence supporting their occurrence (Avila-Pires, 1995; Silva, 1995; Werneck et al., 2012; Bueno et al., 2017).

In this study, we conducted extensive sampling of *Qualea grandiflora* Mart (Vochysiaceae), a widely distributed tree species occurring both in the Cerrado core and Amazonian savannas. We investigated the effects of past climatic changes on the genetic diversity, dynamics of species range and historical connections between the Amazonian savannas and Cerrado core. We tested the hypothesis that during the Pleistocene, there was variation in range toward the north and south of a climatically stable area in the Cerrado core during cooling and warming periods, respectively. Additionally, we tested the existence of corridors connecting the Cerrado core and Amazonian savannas. Specifically, we aimed at answering the following questions: (1) When and from where did the lineages of *Q. grandiflora* disperse? (2) What is the historical evolutionary relationship between the Cerrado core and disjoint Central and western Amazonian savannas? (3) By what mechanism would these regions have been connected and when would these connections have been established?

## Materials and methods

### Studied species, population sampling, and DNA isolation

The studied species, *Q. grandiflora*, belongs to the family Vochysiaceae, occurring exclusively in tropical regions (Vianna, 2006). It is a common species in the Cerrado, observed in 85% of sampled areas in a study on the floristic composition of the Cerrado, conducted by Ratter et al. (2003). It is an arboreal shrub (7–12 m tall) with yellow flowers pollinated mainly by hawk moths and wind-dispersed seeds (Lorenzi, 1992; Oliveira et al., 2004). Because of its widespread yet exclusive distribution in the Cerrado, *Q. grandiflora* can be considered a marker of this biome. Similar to other Vochysiaceae species in the Cerrado, it is an aluminum-accumulating species (Haridasan, 1982) adapted to survive in well-drained acidic soils with high aluminum (Al) saturation and low availability of nutrients and cations (Ca^2+^ and Mg^2+^) (de Andrade et al., 2011).

**Figure 1:**
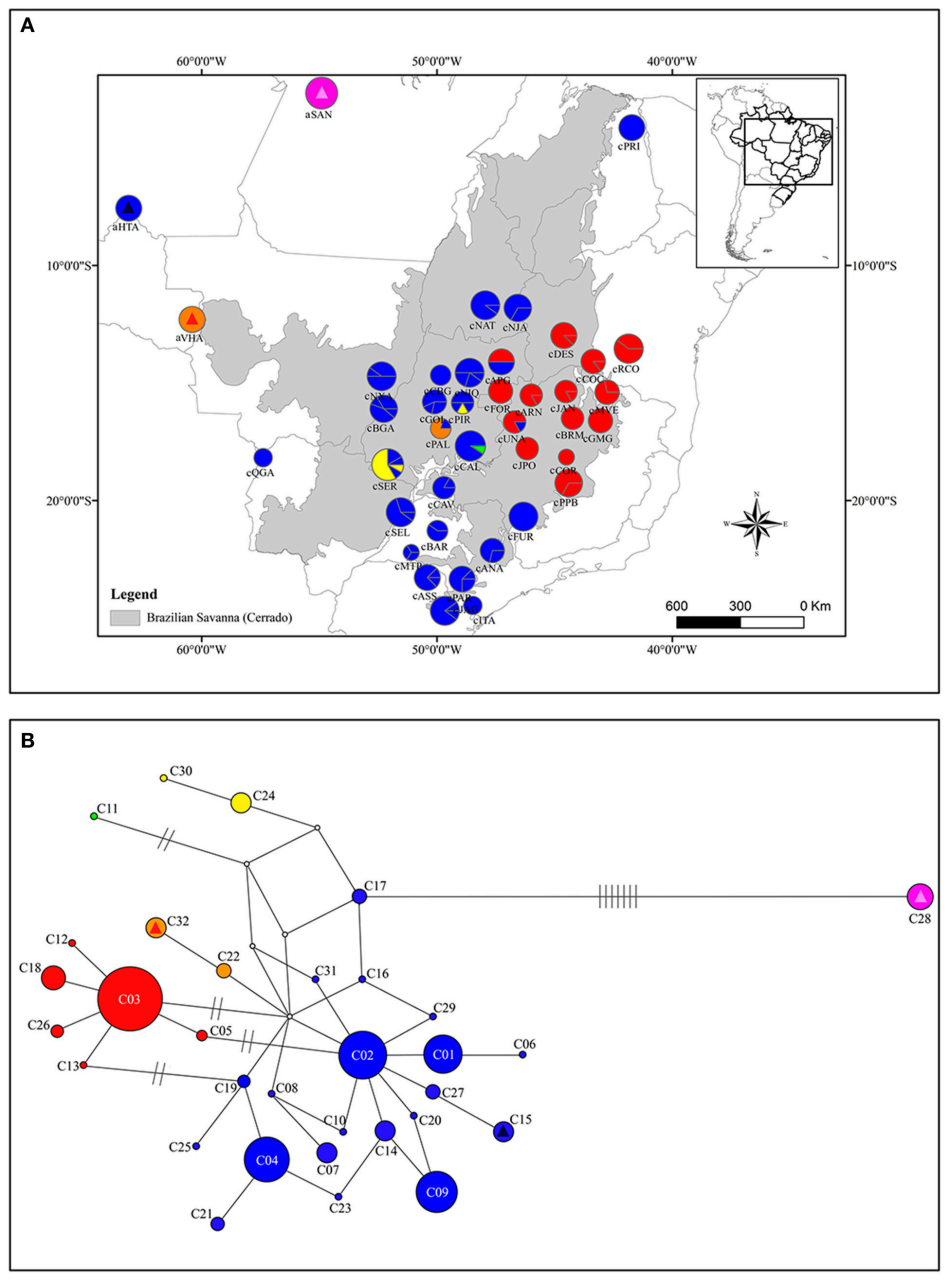
Geographical distribution of cpDNA haplotypes over sampled area for *Qualea grandiflora* **(A)** and Median-joining network showing the relationships between *Qualea grandiflora* haplotypes based on concatenated cpDNA sequences **(B)**. The map and network colors are the same and the size of the circles in **(A,B)** is proportional to the number of individuals. Dashes represent the number of mutations separating the haplotypes. Absence of dashes denotes only one mutational step between haplotypes. The triangles represent the haplotypes that are exclusive from Amazonian enclaves and the squares the haplotypes shared with Cerrado core. Each exclusive or shared haplotype has one triangle or square color.

To map the sites of *Q. grandiflora* occurrence and sample the most of its natural distribution, we conducted an investigation on phytosociological studies and databases of species occurrence (Supplementary Figure 1). Then, 40 populations were sampled between 2°32′S and 24°10′S and between 41°43′W and 63°03′W, from Cerrado core and Amazonian savanna (Table 1 and Figure 1A). Populations located at Cerrado core and Amazonian savanna were named with initial letters “c” and “a,” respectively. Young leaves of adult individuals were collected, dried over silica gel and stored at −20°C. Voucher specimens of the sampled populations were deposited at the herbaria of the Departamento de Botânica da Universidade Federal de Minas Gerais (BHCB) or the Universidade Estadual Paulista (HRCB) (Supplementary Table 1). Genomic DNA was isolated using the cetyltrimethylammonium bromide (CTAB) method (Doyle and Doyle, 1987). We assessed the quantity and quality of DNA by visualization on a 1% agarose gel and using a NanoDrop® spectrophotometer (Thermo Fisher Scientific, Waltham, MA, USA).

**Table 1:** Locations of *Qualea grandiflora* populations in the Brazilian Cerrado biome, genetic diversity indices and distribution of chloroplast DNA (cpDNA) and nuclear DNA (nDNA) haplotypes.

				**cpDNA data**				**nDNA data**			
**Locality/State^a^ (population code)**	**Latitude (S)**	**Longitude (W)**	**Altitude (m)**	***N***	***h***	**π**	**Haplotypes**	***N***	***h***	**π**	**Haplotypes**
Alto do Paraíso de Goiás, GO (cAPG)	14° 06′ 51′′	47° 31′ 27′′	1276	8	0.514	0.0017	C03, C04	–	–	–	–
Amolar, MS (cQGA)	18° 10′ 43′′	57° 23′ 25′′	–	4	0.000	0.0000	C27	–	–	–	–
Analândia, SP (cANA)	22° 07′ 41′′	47° 39′ 03′′	705	7	0.476	0.0004	C01, C02	–	–	–	–
Arinos, MG (cARN)	15° 56′ 00′′	45° 59′ 50′′	514	6	0.333	0.0002	C03, C05	–	–	–	–
Assis, SP (cASS)	22° 34′ 38′′	50° 24′ 50′′	533	8	0.464	0.0005	C01, C02, C06	–	–	–	–
Barbosa, SP (cBAR)	21° 17′ 10′′	49° 59′ 02′′	404	5	0.600	0.0004	C01, C02	–	–	–	–
Barra do Garças, MT (cBGA)	15° 51′ 17′′	52° 15′ 55′′	577	9	0.750	0.0015	C07, C08, C09, C10	4	0.857	0.0067	N01, N02, N03, N04, N05
Brasília de Minas, MG (cBRM)	16° 09′ 26′′	44° 14′ 31′′	–	6	0.000	0.0000	C03	–	–	–	–
Caldas Novas, GO (cCAL)	17° 40′ 32′′	48° 45′ 12′′	685	11	0.182	0.0009	C09, C11	5	0.933	0.0074	N02, N05, N06, N07, N08, N09, N10, N11
Campina Verde, MG (cCAV)	19° 27′ 14′′	49° 42′ 29′′	646	6	0.333	0.0005	C02, C09	–	–	–	–
Cocos, BA (cCOC)	14° 05′ 01′′	44° 31′ 04′′	671	7	0.286	0.0002	C03, C12	1	1.000	0.0017	N07, N12
Corinto, MG (cCOR)	18° 22′ 39′′	44° 30′ 09′′	662	3	0.000	0.0000	C03	–	–	–	–
Corumbá de Goiás, GO (cCBG)	15° 54′ 01′′	48° 53′ 13′′	1082	5	0.000	0.0000	C09	–	–	–	–
Formosa, GO (cFOR)	15° 21′ 07′′	47° 25′ 45′′	667	7	0.000	0.0000	C03	3	0.933	0.0015	N07, N08, N13, N14, N15
Furnas, MG (cFUR)	20° 41′ 00′′	46° 19′ 37′′	749	10	0.000	0.0000	C01	3	1.000	0.0080	N02, N07, N16, N17, N18, N19
Goiás, GO (cGOI)	15° 59′ 17′′	50° 06′ 36′′	587	7	0.667	0.0011	C04, C09, C14	–	–	–	–
Grão Mogol, MG (cGMG)	16° 32′ 31′′	43° 03′ 05′′	875	7	0.000	0.0000	C03	–	–	–	–
Humaitá, AM (aHTA)	7° 34′ 01′′	63° 06′ 11′′	63	8	0.000	0.0000	C15	6	0.621	0.0046	N20, N21, N22
Itararé, SP (cITA)	24° 05′ 10′′	49° 12′ 32′′	693	4	0.000	0.0000	C02	–	–	–	–
Jaguariaíva, PR (cJAG)	24° 10′ 41′′	49° 40′ 08′′	905	10	0.378	0.0004	C01, C02, C16	–	–	–	–
Januária, MG (cJAN)	15° 21′ 46′′	44° 31′ 19′′	622	6	0.333	0.0002	C03, C05	–	–	–	–
João Pinheiro, MG (cJPO)	17° 46′ 04′′	46° 10′ 05′′	800	6	0.000	0.0000	C03	–	–	–	–
Martinópolis, SP (cMTP)	22° 12′ 27′′	51° 05′ 53′′	448	3	1.000	0.0020	C01, C02, C17	2	1.000	0.0019	N07, N08, N23, N24
Mato Verde, MG (cMVE)	15° 23′ 34′′	42° 46′ 22′′	1013	7	0.476	0.0004	C03, C18	–	–	–	–
Natividade, TO (cNAT)	11° 41′ 38′′	47° 42′ 07′′	675	10	0.200	0.0001	C04, C19	4	0.929	0.0019	N07, N11, N14, N15, N25, N26
Niquelândia, GO (cNIQ)	14° 44′ 09′′	48° 36′ 48′′	539	10	0.733	0.0014	C04, C09, C19, C20	3	0.800	0.0034	N13, N27, N28, N29
Nova Xavantina, MT (cNXA)	14° 42′ 53′′	52° 21′ 14′′	334	10	0.644	0.0009	C07, C09, C14	2	1.000	0.0025	N07, N30
Novo Jardim, TO (cNJA)	11° 48′ 28′′	46° 34′ 05′′	604	9	0.500	0.0004	C04, C21	–	–	–	–
Palmeira de Goiás, GO (cPAL)	16° 46′ 48′′	49° 50′ 44′′	647	5	0.400	0.0009	C04, C22	4	0.964	0.0013	N02, N09, N31, N32, N33, N34, N35
Paranapanema, SP (cPAR)	23° 20′ 35′′	48° 55′ 56′′	658	8	0.607	0.0011	C01, C02, C17	5	0.956	0.0083	N01, N02, N05, N10, N36, N37, N38, N39
Paraopeba, MG (cPPB)	19° 15′ 28′′	44° 24′ 10′′	745	9	0.500	0.0004	C03, C26	1	1.000	0.0085	N40, N41
Pirenópolis, GO (cPIR)	15° 50′ 21′′	48° 54′ 46′′	954	6	0.800	0.0026	C09, C23, C24, C25	1	1.000	0.0017	N05, N10
Piripiri, PI (cPRI)	4° 08′ 24′′	41° 43′ 07′′	228	8	0.000	0.0000	C04	5	0.867	0.0026	N13, N14, N26, N42, N43
Rio de Contas, BA (cRCO)	13° 32′ 31′′	41° 51′ 23′′	1148	10	0.533	0.0004	C03, C18	–	–	–	–
Santarém, PA (aSAN)	2° 32′ 12′′	54° 54′ 13′′	54	12	0.000	0.0000	C28	4	0.857	0.0035	N11, N13, N44, N45, N46
São Desidério, BA (cDES)	12° 58′ 52′′	44° 36′ 42′′	654	8	0.250	0.0002	C03, C13	–	–	–	–
Selvíria, MS (cSEL)	20° 29′ 52′′	51° 32′ 41′′	361	10	0.600	0.0008	C01, C02, C17	7	0.989	0.0068	N02, N25, N28, N36, N47, N48, N49, N50
											N51, N52, N53, N54, N55
Serranópolis, GO (cSER)	18° 28′ 32′′	52° 05′ 42′′	735	12	0.667	0.0019	C01, C02, C24, C29, C30	2	1.000	0.0099	N11, N54, N56, N57
Unaí, MG (cUNA)	16° 39′ 31′′	46° 42′ 15′′	636	6	0.333	0.0010	C03, C31	3	0.933	0.0086	N02, N05, N07, N29, N58
Vilhena, RO (aVHA)	12° 17′ 54′′	60° 24′ 31′′	350	8	0.000	0.0000	C32	1	1.000	0.0026	N27, N59

a *Abbreviation of Brazilian States: AM, Amazonas; BA, Bahia; GO, Goiás; MG, Minas Gerais; MS, Mato Grosso do Sul; MT, Mato Grosso; PA, Pará; PI, Piauí; PR, Paraná; SP, São Paulo; TO, Tocantins*.

*N, sample size; h, haplotype diversity; π, nucleotide diversity, – missing data*.

### Amplification and sequencing

Screening for the amplification and polymorphism of chloroplast DNA (cpDNA) regions was conducted using 30 primer pairs for previously described cpDNA regions (Supplementary Table 2) in a subset of samples. The *trn*S*-trn*G and *psb*A*-trn*H regions showed a high number of variable sites, while the other tested regions showed only few or no variation. These regions were amplified for 301 individuals sampled from 40 populations using primers described by Sang et al. (1997) (*psb*A*-trn*H) and Hamilton (1999) (*trn*S*-trn*G) (GenBank accession numbers: MH020057–MH020101; Supplementary Table 3A). Additionally, one single copy nuclear gene (nDNA: AGT1) was amplified for a subset of 65 individuals (130 allele copies) from 20 populations (GenBank accession numbers: MH020102–MH020160; Supplementary Table 3B). Polymerase chain reactions (PCR) were conducted as described in the supplementary data (Supplementary Materials and Methods). Consensus sequences were obtained using the package Phred/Phrap/Consed (Ewing and Green, 1998; Ewing et al., 1998; Gordon et al., 1998), aligned using MUSCLE in MEGA 6 (Tamura et al., 2011) and adjusted manually.

### Haplotype identification, genetic diversity, and population structure analyses

The haplotypes of the two concatenated cpDNA regions were defined by analyzing the sequences using DnaSP 5.10 (Librado and Rozas, 2009). The inversion and all insertion/deletions (indels) observed were considered fifth character states and coded as single mutations, regardless of their size. The nDNA haplotypes were reconstructed using PHASE in DnaSP 5.10 (Librado and Rozas, 2009), using 10,000 iterations and 1,000 of them deleted as burn-ins. Only haplotypes recovered with a posterior probability of 0.90 were considered.

The overall *F*_ST_ was evaluated using analysis of molecular variance (AMOVA) with ARLEQUIN 3.5 (Excoffier and Lischer, 2010). Genetic clustering was inferred using GENELAND package in R (Guillot et al., 2005a,b; R Development Core Team, 2016). The analysis was performed using two cpDNA regions concatenated as a single sequence and nDNA haplotypes encoded as alleles. We carried out the spatialized and the non-spatialized analyses in order to verify the influence of geographical distance in the clustering. We choose the uncorrelated allele frequencies model and made 10 parallel runs with 1 × 10^6^ iterations each with a thinning of 1 × 10^3^ for both analyses. We set *K* as varying between 1 and 20. To generate the map of probability of population membership, we performed another run with 1 × 10^6^ iterations and a thinning of 1 × 10^3^ with *K* fixed in the number of clusters with the highest density on the previous analyses.

The location of potential barriers to gene flow was inferred using Monmonier's maximum difference algorithm (Monmonier, 1973) with BARRIER 2.2 (Manni et al., 2004). The Mantel test (Mantel, 1967), as implemented in ARLEQUIN 3.5 (Excoffier and Lischer, 2010), was employed to evaluate the relationship between genetic and geographical distances for both DNA regions. For barrier inferences and Mantel test we used the Slatkin's linearized distance [*F*_ST_/(1–*F*_ST_)].

After defining the genetic clusters, genetic diversity indices (nucleotide diversity, π and haplotype diversity, *h*) for cpDNA and nDNA were estimated for each cluster, each population and all populations together for both regions (cpDNA and nDNA) using ARLEQUIN 3.5 (Excoffier and Lischer, 2010).

### Demographic analyses

Recent population expansion was tested for cpDNA locus for all populations and each cluster using mismatch analysis with ARLEQUIN 3.5 (Excoffier and Lischer, 2010). For the mismatch analyses, 10,000 parametric bootstrap replicates were carried out. Neutrality tests such as Tajima's D (Tajima, 1989) and Fu's *F*s (Fu, 1997) were performed using both cpDNA and nDNA separately. These tests suggest a demographic expansion scenario when significant negative values are obtained. Additionally, we modeled the changes in effective population size for the species over continuous time in a multilocus context using the Extended Bayesian Skyline Plot (EBSP) model with BEAST 2 (Heled and Drummond, 2008; Bouckaert et al., 2014). For this analysis, we used only the individuals with complete datasets (i.e., both cpDNA and nDNA loci sequenced, treated as independent gene trees). We established a strict clock model for the cpDNA gene tree, with the substitution rate fixed as 3 × 10^−3^ substitutions per site per million years (s s^−1^ Myr^−1^; Buzatti et al., 2017). The clock model for the nDNA gene tree was established as a relaxed lognormal model, with the substitution rate inferred in relation to that of cpDNA. The molecular clock models were stablished from standard deviation of uncorrelated lognormal relaxed clock (ucld.stdev) parameter. According to Bouckaert et al. (2014), only if the estimated value of this parameter is lower than one, the strict molecular clock can be adopted. We performed two independent runs with 1 × 10^8^ generations each and 10^4^ thinning. The stationarity and the convergence of the independent runs were checked with Tracer 1.6 (Rambaut and Drummond, 2013a), yet with a burn-in of 5% (Supplementary Figure 2).

### Phylogenetic relationships among haplotypes

The phylogenetic relationships among haplotypes were inferred using the median-joining algorithm (MJ), based on parsimony criteria (Bandelt et al., 1999), using NETWORK 4.6.1.2 (fluxus-engineering.com) and Bayesian inference (BI) performed using BEAST 2 (Bouckaert et al., 2014), considering cpDNA and nDNA regions separately. To infer relationships through BI of cpDNA and nDNA haplotypes, we used BEAST 2 (Bouckaert et al., 2014). The BEAST input files were created with BEAUTi 2.0.2 (Bouckaert et al., 2014) using coalescent constant population tree prior process as well as GTR+I and GTR+G+I substitution models for cpDNA and nDNA regions, respectively. The evolutionary model was selected previously using the Akaike Information Criterion (AIC; Kelchner and Thomas, 2007) with jModelTest 2.1.5 (Guindon and Gascuel, 2003; Darriba et al., 2012). We used the substitution rate estimated by Buzatti et al. (2017) for the family Vochysiaceae for cpDNA (3 × 10^−3^ s s^−1^ Myr^−1^) and a general substitution rate for the AGT1 region (5 × 10^−3^ s s^−1^ Myr^−1^; Blanco-Pastor et al., 2012), under strict and relaxed lognormal clock models, respectively. The BEAST analysis was run for 30 million generations and sampled every 1000th generation. Convergence between chains and effective sample sizes (ESS > 200) were checked with Tracer 1.6 (Rambaut and Drummond, 2013a). All trees obtained prior to convergence were discarded and trees were summarized in a Maximum Clade Credibility (MCC) tree using TreeAnnotator 2.1.2 (Rambaut and Drummond, 2013b). The final tree was viewed using FigTree 1.4.3 (Rambaut, 2016).

### Species colonization history

To gain insights about the historical colonization of *Q. grandiflora*, we reconstructed a continuous spatiotemporal model for cpDNA using the Bayesian approach with BEAST 1.8.4 (Drummond et al., 2012). As we expected a strict molecular clock for our cpDNA data (see above), we modeled a time-homogeneous Relaxed Random Walk (RRW) (Lemey et al., 2010) to infer the lineage colonization routes. Firstly, we performed exploratory analyses using the entire dataset; however, these analyses showed low ESS for several parameters and did not reach convergence, probably because of an excess of copies of more frequent haplotypes. Thence, we subsampled 185 sequences, maintaining the haplotype proportions and including at least one copy of each distinct haplotype by locality. As the Brownian diffusion model has poor performance when not all sequences are associated with unique locations, the analysis was performed with noise introduction in samples with identical coordinates using the “jitter” option, set as 0.75. The substitution model for the reduced dataset was TVM + I. Additionally, we used the same substitution rate as that in the BI analysis to calibrate the diffusion events. Each analysis was performed twice using distinct random seeds with 2 × 10^8^ generations and 10^4^ thinning each. The maximum credibility tree was annotated using TreeAnnotator 2.1.2 (Rambaut and Drummond, 2013b) and this tree was submitted to SPREAD 1.0.7 (Bielejec et al., 2011) to generate a visual representation of the spatial diffusion patterns. Finally, we created the graphic representation of the major events in certain distinct time slices.

### Ecological niche modeling

Ecological niche modeling (ENM) of *Q. grandiflora* was performed using Maxent 3.4.1 (Phillips et al., 2017) with 440 species occurrences registered in the Cerrado (Supplementary Figure 1) extracted from the NeoTropTree database (Oliveira-Filho, 2017) and 40 points of sampled populations (Supplementary Figure 1). To determine the palaeodistribution of *Q. grandiflora*, we made projections of species occurrence suitability in the current (0 ka; pre-industrial), Last Glacial Maximum (LGM; 21 ka) and Last Interglacial (LIG; 130 ka) time periods based on climatic simulations (Hijmans et al., 2005). Palaeoclimatic data represent climate data downscaled from simulations using Global Climate Models (GCMs) based on the Coupled Model Intercomparison Project Phase 5 (CMIP5; Taylor et al., 2012). For the LIG model, we used the approach of Otto-Bliesner et al. (2006), while for LGM, we employed the Community Climate System Model, CCSM4 (Gent et al., 2011). All geographic information system (GIS) analyses were performed using ArcGIS 10 (ESRI, 2011).

Environmental data (19 standard BIOCLIM variables) were obtained for all geographical coordinates, at a spatial resolution of 1 km, from the WorldClim database (Hijmans et al., 2005). The bioclimatic layers spanning from 12°47′N to 34°46′S and from 78°31′W to 35°00′W (spatial range larger than that of the Cerrado) were cropped according to Buzatti et al. (2017). Of the 19 bioclimatic variables, ten were used in the distribution models; to avoid over-parameterization of our modeling, we eliminated variables that correlated strongly (*r* > 0.9) (Dormann et al., 2013; Bünger et al., 2016; Bueno et al., 2017; Buzatti et al., 2017). The finally selected variables were annual mean temperature (BIO1), mean diurnal temperature range (BIO2), isothermality (BIO3), temperature annual range (BIO7), mean temperature of warmest quarter (BIO10), mean temperature of coldest quarter (BIO11), annual precipitation (BIO12), precipitation of driest month (BIO14), precipitation seasonality (BIO15), and precipitation of driest quarter (BIO17).

For predicting potential areas of climate stability throughout the Quaternary Period for *Q. grandiflora*, we used similar protocols of recent studies on several Neotropical biomes (Carnaval and Moritz, 2008; Werneck, 2011; Werneck et al., 2012; Bueno et al., 2017; Buzatti et al., 2017), wherein converted models were produced from continuous outputs into presence/absence maps by setting the lowest presence threshold for each model. This approach maximizes the agreement between observed and modeled distributions, balancing the cost arising from an incorrect prediction against the benefit gained from the correct prediction (Pearson, 2006). A map of areas showing historical stability was generated by summing the presence/absence maps obtained for the current conditions, LIG and LGM projections. This combined map depicted areas potentially occupied by *Q. grandiflora* during the climatic oscillations in the Quaternary. These historically stable areas called refuges are defined as the grid cells for which the presence of the species was inferred in all time projections.

To calibrate and evaluate models quality, we divided the data into a training set (75% of occurrences) and test or validation set (25% of occurrences). We constructed models 10 times and averaged the output to produce data used in the downstream analyses. Next, we defined a threshold value above grid cells, considered to have environmental characteristics suitable for maintaining viable populations of the species (Pearson, 2006). We used “minimum training presence” as the threshold selection method because it assumes that species presence is restricted to sites at least as suitable as those where the species has been observed thus far (Pearson, 2006).

For validating the models produced, we used the precision measures of sensitivity, specificity and True Skill Statistic (TSS) as threshold-dependent accuracy measures and Area Under Curve (AUC) as a threshold-independent measure (Allouche et al., 2006; Liu et al., 2009). For calculating these parameters beyond the *Q. grandiflora* points, we used 202 occurrences of *Eugenia uruguayensis* obtained from NeoTropTree (Oliveira-Filho, 2017). The *E. uruguayensis* points were used to represent a typical environment wherein there are no *Q. grandiflora* records. Thus, a robust model distinguishes clearly between presence and absence and would have high sensitivity and specificity (Peterson et al., 2011).

## Results

### Genetic diversity and phylogeographic structure

The alignment of cpDNA regions produced fragments of 664 bp (*psb*A*-trn*H) and 693 bp (*trn*S*-trn*G). In the concatenated form, the entire sequence (1,357 bp) showed 29 polymorphic sites, specifically 14 insertions/deletions (indels, ranging from six to 30 bp), one inversion (with 30 bp) and 14 substitutions, generating 32 haplotypes. The nuclear gene sequence (nDNA: AGT1) was 1,182 bp long and highly variable and showed 64 variable sites, all substitutions, generating 59 haplotypes.

**Figure 2:**
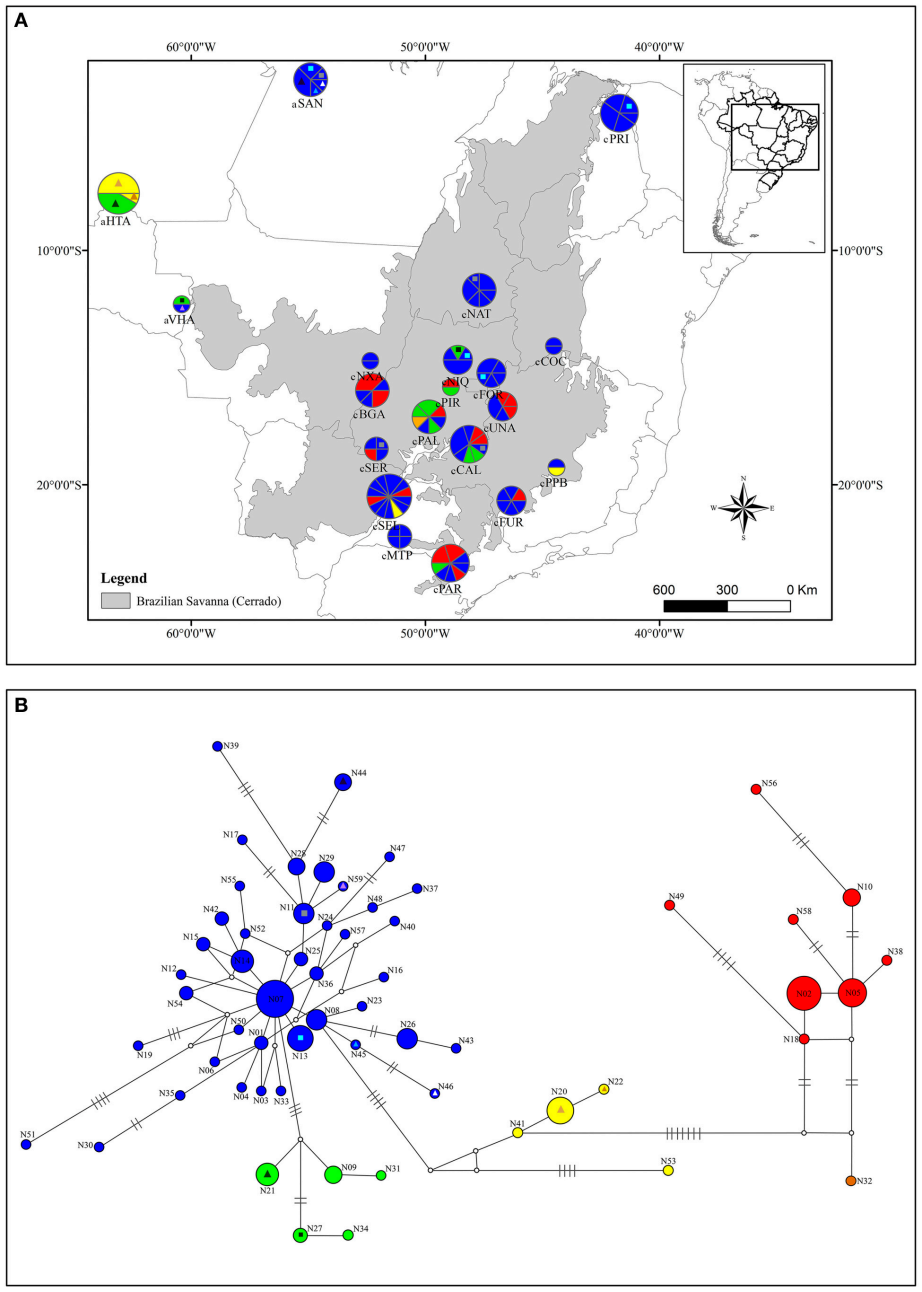
Geographical distribution of nDNA haplotypes over sampled area for *Qualea grandiflora* **(A)** and Median-joining network showing the relationships between *Qualea grandiflora* haplotypes based on nDNA region **(B)**. The map and network colors are equivalent and the size of the circles in **(A,B)** is proportional to the number of individuals. Dashes represent the number of mutations separating the haplotypes. Absence of dashes denotes only one mutational step between haplotypes. The triangles represent the haplotypes that are exclusive from Amazonian enclaves and the squares the haplotypes shared with Cerrado core. Each exclusive or shared haplotype has one triangle or square color.

The molecular diversity indices and haplotype distribution in each population for both datasets are summarized in Table 1. Of the 40 populations analyzed for cpDNA, 27 had two or more haplotypes, while 13 had no genetic diversity (Table 1). Populations aHTA, aVHA, cQGA, aSAN, and cPRI, located in the Amazonian savannas or peripheral areas, were monomorphic. The central portion of the Cerrado core harbors most of the sampled diversity for cpDNA (Figure 1A) and populations cPIR, cNIQ and cBGA, located in this area, had the highest genetic diversity indices (Table 1). All populations were polymorphic for nDNA, with two or more haplotypes (Table 1, Figure 2A). In the same way, the mean of diversity indices to nDNA were lower in the Amazon savannas and peripheral areas (*h*_*mean*_± S.D. = 0.836 ± 0.158; π_*mean*_ ± S.D. = 0.003 ± 0.001) than in the Cerrado core populations (*h*_*mean*_± S.D. = 0.951 ± 0.061; π_*mean*_ ± S.D. = 0.005 ± 0.003). The total haplotype diversity was 0.888 and 0.973 and total nucleotide diversity was 0.0027 and 0.0071 for cpDNA and nDNA, respectively. The populations showed high genetic differentiation for cpDNA (*F*_ST_ = 0.790); however, for nDNA, this differentiation was lower (*F*_ST_ = 0.231). Genetic distance correlated significantly with geographical distance for cpDNA (*r* = 0.039, *P* = 0.000), while for nDNA, these distances did not correlate (*r* = −0.042, *P* = 0.47).

**Figure 3:**
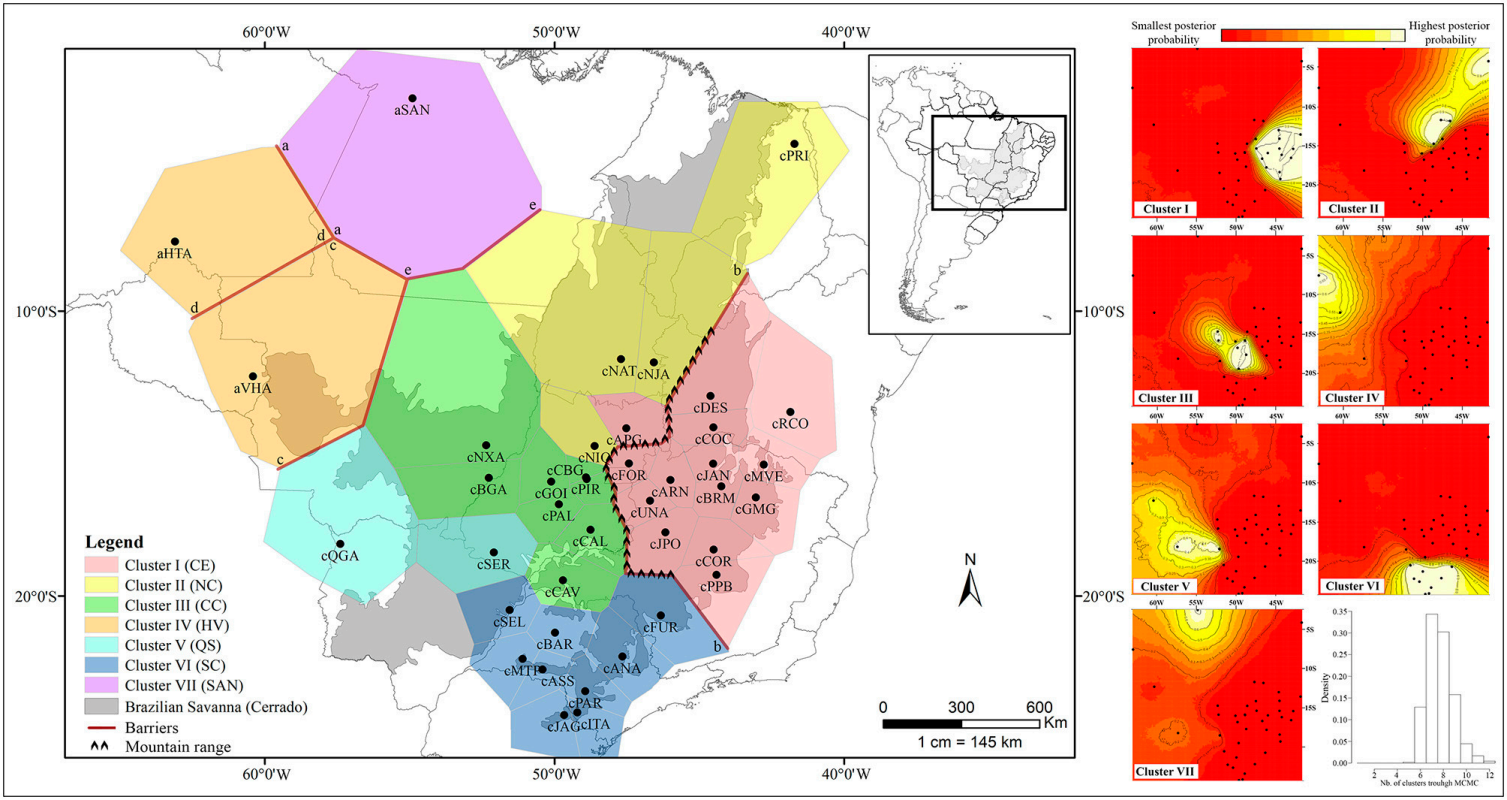
Population structure of *Qualea grandiflora* determined by Bayesian Analysis using GENELAND based on nDNA and concatenated cpDNA sequences. Putative barriers were identified using Monmonier's algorithm with BARRIER based on concatenated cpDNA sequences (on the **Left**). The maps of posterior probabilities of population membership generated by GENELAND are shown on the **Right**. Barplot shows the density in relation to the number of clusters through Markov Chain Monte Carlo (MCMC).

The spatial and non-spatial models in GENELAND estimated the number of clusters of *Q. grandiflora* as seven and six, respectively (Figure 3 and Supplementary Figure 3). Exclusive haplotypes were observed in all clusters (Table 2) for both DNA regions, indicating a genetic structuring. Clusters III (CC) and V (QS) possessed the highest genetic diversities (Table 2). The haplotype distributions of the cpDNA and nDNA support the isolation of Amazonian savannas, with high occurrence of exclusive haplotypes in these regions (Figures 1, 2). The major barriers identified using Monmonier's maximum difference algorithm from the cpDNA dataset isolated the Amazonian populations aSAN, aHTA, and aVHA from each other and from the remaining populations, highlighting the isolation of Amazonian savanna populations. The other major barriers separated the cluster CE from the other clusters (Figure 3—left).

**Table 2:** Genetic diversity indices for phylogeographic groups of *Qualea grandiflora* based on chloroplast DNA (cpDNA) data and for all populations based on cpDNA and nuclear DNA (nDNA) data.

	**Phylogeographyc groups^a^**	**Sample**	**Total of haplotypes**	**Shared haplotypes**	**Exclusive haplotyopes**	***h***	**π**
cpDNA	Cluster I (CE)	96	8	C03, C04	C05, C12, C13, C18, C26, C31	0.409	0.0006
	Cluster II (NC)	37	5	C04, C09	C19, C20, C21	0.422	0.0006
	Cluster III (CC)	59	12	C02, C04, C09, C24	C07, C08, C10, C11, C14, C22, C23, C25	0.729	0.0016
	Cluster IV (HV)	16	2	–	C32, C15	0.533	0.0020
	Cluster V (QS)	16	6	C01, C02, C24	C27, C29, C30	0.767	0.0024
	Cluster VI (SC)	65	5	C01, C02	C06, C16, C17	0.578	0.0005
	Cluster VII (SAN)	12	1	–	C28	0.000	0.0000
	All populations	301	32	–	–	0.888	0.0027
nDNA	Cluster I (CE)	16	12	N02, N05, N07, N08, N13, N14, N15, N29	N12, N40, N41, M58	0.942	0.0060
	Cluster II (NC)	24	12	N07, N11, N13, N14, N15, N25, N27, N28, N29	N26, N42, N43	0.928	0.0028
	Cluster III (CC)	30	17	N01, N02, N05, N07, N08, N10, N11	N03, N04, N06, N09, N30, N31, N32, N33, N34, N35	0.945	0.0080
	Cluster IV (HV)	14	5	N27	N20, N21, N22, N59	0.725	0.0052
	Cluster V (QS)	4	4	N11, N54	N56, N57	1	0.0099
							
	Cluster VI (SC)	34	22	N01, N02, N05, N07, N08, N10, N25, N28	N16, N17, N18, N19, N23, N24, N36, N37, N38, N39, N47, N48, N49, N50	0.977	0.0078
	Cluster VII (SAN)	8	5	N11, N13	N44, N45, N46,	0.893	0.0035
	All populations	130	59	–	–	0.973	0.0071

a *The phylogeographic groups are composed by following populations: CE: cARN, cBRM, cCOC, cCOR, cDES, cFOR, cGMG, cJAN, cJPO,cMVE, cPPB, cRCO, cUNA, cAPG; NC: cNAT, cNIQ, cNJA, cPRI; CC: cBGA, cCAL, cCAV, cCBG, cGOI, cNXA, cPAL, cPIR; HV: aHTA, aVHA; QS: cQGA, cSER, SC: cANA, cASS, cBAR, cFUR, cITA, cJAG, cMTP, cPAR, cSEL; SAN: aSAN*.

*h, haplotype diversity; π, nucleotide diversity, – absent*.

### Haplotype relationships

The relationships among the haplotypes across the two methods used (MJ network—Figures 1B, 2B and BI analyses—Supplementary Figures 4, 5) showed concordant results. The network for the cpDNA dataset revealed no common central haplotype and many reticulations indicating equiprobable evolutionary paths (Figure 1B). Despite these reticulations, it was possible to identify the lineages (in this study, each lineage was considered a haplotype group separated by at least two mutational steps from the other groups) showing a phylogeographic structure concordant with the GENELAND clusters. Two lineages were widely distributed in the Cerrado, one in central eastern Cerrado, and the other in most of the Cerrado except in populations aSAN and aVHA and central eastern Cerrado (Figure 1). The haplotype of population aSAN (C28) was clearly separated from those of the remaining ones (seven mutational steps from its closest haplotype). The phylogenetic relationships among the nDNA haplotypes inferred by Bayesian analysis showed several highly supported clades (Supplementary Figure 5), concordant with the MJ network (Figure 2B). The lineage originating from haplotype N07 gave the network a complex star-like shape, suggesting recent expansion (Figure 2B).

### Species colonization history

The values observed in the mismatch distribution tests for cpDNA did not differ from the expected values under the sudden-expansion model (*P* > 0.05), supporting the hypothesis of recent demographic and spatial expansions in all phylogeographic clusters for cpDNA and for all populations together (Table 3). Additionally, the neutrality test Fu's *F*s detected population expansion with nDNA; however, Tajima's D did not reveal any expansion for both data sets (Table 3). Overall, EBSP did not detect population size shifts over time as well (Supplementary Figure 6A), although there was evidence for a subtle change in the most recent coalescent events of the species (Supplementary Figure 6B). The inconsistence of these results could be related to a limited dispersion syndrome (anemochory). According to Excoffier et al. (2009) and Arenas et al. (2012) populations can seem to be stationary in the expansion analysis if they have low dispersion ability associated with recent range expansion history.

**Table 3:** Mismatch distribution analysis (parameters of demographic and spatial expansion) of phylogeographic groups of *Qualea grandiflora* using chloroplast DNA (cpDNA) data and of all populations using cpDNA and nuclear DNA (nDNA) data.

		**Mismatch analysis**				**Neutrality tests**	
		**Demographic expansion**		**Spatial expansion**		**Tajima's D (*P*-value)**	**Fu's Fs (*P*-value)**
	**Phylogeographyc groups^a^**	**SSD (*P*-value)**	**Raggedness (*P*-value)**	**SSD (*P*-value)**	**Raggedness (*P*-value)**		
cpDNA	Cluster I (CE)	0.078 (0.121^b^)	0.235 (0.253^b^)	0.027 (0.180^b^)	0.235 (0.394^b^)	−0.015 (0.828)	0.590 (0.488)
	Cluster II (NC)	0.103 (0.164^b^)	0.209 (0.240^b^)	0.020 (0.174^b^)	0.209 (0.385^b^)	0.258 (0.967)	0.238 (0.432)
	Cluster III (CC)	0.109 (0.113^b^)	0.406 (0.286^b^)	0.061 (0.196^b^)	0.406 (0.486^b^)	−0.397 (0.470)	1.010 (0.676)
	Cluster IV (HV)	–	–	–	–	–	–
	Cluster V (QS)	–	–	–	–	–	–
	Cluster VI (SC)	0.038 (0.315^b^)	0.211 (0.447^b^)	0.075 (0.100^b^)	0.340 (0.386^b^)	−0.350 (0.650)	0.173 (0.306)
	Cluster VII (SAN)	–	–	–	–	–	–
	All populations	0.069 (0.176^b^)	0.234 (0.315^b^)	0.032 (0.185^b^)	0.235 (0.407^b^)	−0.106 (0.757)	0.481 (0.343)
nDNA	Cluster I (CE)	Na	Na	Na	Na	0.338 (0.856)	0.354 (0.381)
	Cluster II (NC)	Na	Na	Na	Na	−0.134 (0.477)	0.414 (0.407)
	Cluster III (CC)	Na	Na	Na	Na	0.308 (0.807)	0.145 (0.403)
	Cluster IV (HV)	Na	Na	Na	Na	1.043 (0.996)	4.049 (0.766)
	Cluster V (QS)	Na	Na	Na	Na	−0.721 (0.272)	0.512 (0.371)
	Cluster VI (SC)	Na	Na	Na	Na	0.160 (0.601)	−2.092 (0.149)
	Cluster VII (SAN)	Na	Na	Na	Na	–	–
	All populations	Na	Na	Na	Na	−0.905 (0.192)	−24.591 (0.001)

a *The phylogeographic groups are composed by following populations: CE: cARN, cBRM, cCOC, cCOR, cDES, cFOR, cGMG, cJAN, cJPO,cMVE, cPPB, cRCO, cUNA, cAPG; NC: cNAT, cNIQ, cNJA, cPRI; CC: cBGA, cCAL, cCAV, cCBG, cGOI, cNXA, cPAL, cPIR; HV: aHTA, aVHA; QS: cQGA, cSER, SC: cANA, cASS, cBAR, cFUR, cITA, cJAG, cMTP, cPAR, cSEL; SAN: aSAN*.

b *P > 0.05, which means that the population set (either groups or the whole species) mismatch distribution did not differ significantly from a sudden-expansion model*.

*Na, not applicable for nDNA*.

*−, not possible to estimate*.

**Figure 4:**
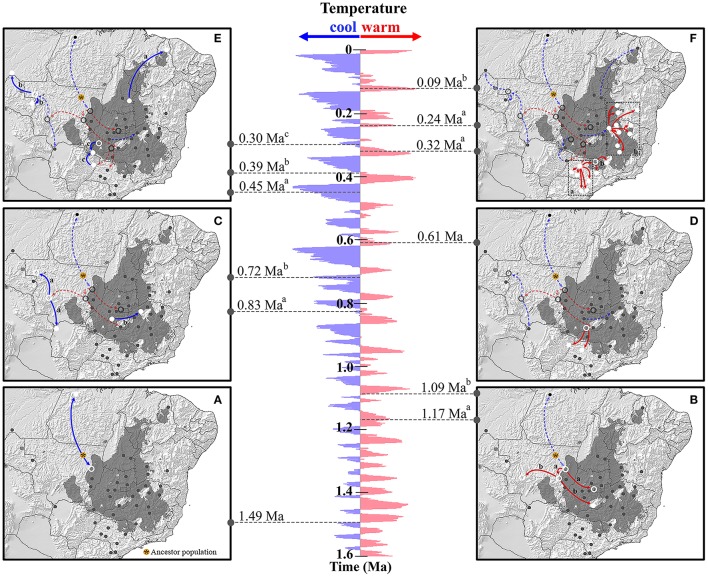
Spatio-temporal dynamics of lineage colonization from ancestral population to 40 populations sampled in the Cerrado core and Amazonian savannas based on Relaxed Random Walk. The map shows the stable areas inferred by Ecological Niche Modeling. Arrows between locations indicate branches of the Bayesian tree along which the relevant lineage colonization occurred. The pannels are arranged from the most ancient colonization event **(A)** to the most recent colonization event **(F)**. The map is a schematic representation of kml file generated using SPREAD and visualized using Google Earth (http://earth.google.com). The δ^18^O curve corresponds to the composite benthic stable oxygen isotope ratios obtained from Lisiecki and Raymo (2005).

The RRW model suggested that *Q. grandiflora* lineage colonization (Figure 4 and Supplementary Figure 7) began in the Early Pleistocene (ca 1.49 Ma), with the most probable ancestral location in central-west Brazil. The first colonization events occurred from this region in two directions, toward the north (aSAN in the Amazonian savanna) and toward the south (Figure 4A). At approximately 1.17 Ma, the area occupied by *Q. grandiflora* expanded toward central Cerrado area (cPIR population; Figure 4B) and to a region located next to the western boundary of the Cerrado. The subsequent events (1.09 Ma and 830 ka) enabled the colonization of regions at the extreme western boundaries of the Cerrado (cQGA population; Figures 4B,C). At 450 ka, lineages dispersed toward north-eastern Brazil (cPRI population, Figure 4E) and later, at approximately 390 ka, toward north-western Brazil (aHTA and aVHA populations; Figure 4E). Colonization toward the Amazonian savannas (aSAN and aHTA) and boundary area between north-western Cerrado and Amazon forest (aVHA) occurred during glacial or cooling periods.

Colonization to southern Cerrado began at ca 610 ka, in an interglacial or warming period and most of the colonization toward this region similarly occurred in a warming time slice (mainly at 320 and 240 ka; Figure 4F). At 300 ka, in a cooling period, lineages dispersed to the cSER population region from the northernmost region (next to cPIR population) and from southern Cerrado (Figure 4E). The last major expansion events began at ~120 ka (warming period) when lineages spread across eastern Brazil (Figure 4F).

### Ecological niche modeling

**Figure 5:**
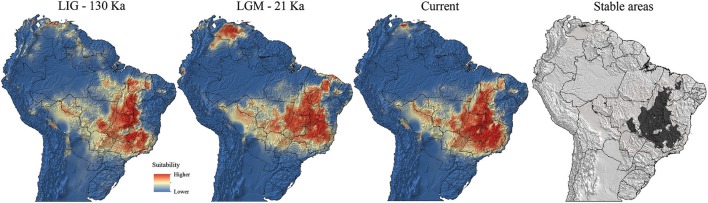
Predicted suitability areas for *Qualea grandiflora* occurrence across Brazil in the Last Interglacial (LIG; 130 ka), Last Glacial Maximum (LGM; 21 ka) and under current climatic conditions (0 ka; pre-industrial) and climatically stable areas.

The ENM indicated significant changes in the environmental suitability areas and distribution ranges of *Q. grandiflora* over the Quaternary (Figure 5). The AUC and TSS values indicated that the generated models performed well to predict species occurrence in relation to the bioclimatic variables (Supplementary Table 4).

The maximum latitudinal expansions of *Q. grandiflora* occurred during warming (LIG and current) periods, with the suitability areas extending from the southernmost part to the northern portion of the Cerrado. Moreover, in the LIG, there was a putative connection between the Amazonian savanna (population aSAN) and Cerrado core (Figure 5). Additionally, the models suggested low suitability of south central Cerrado in the LGM. During this cold period, the high-suitability areas extended toward the west and east. Remarkably, the high-suitability areas advanced northwards and were absent in southern Cerrado (approximately 20°S or beyond) during the LGM. Contrastingly, during the LIG, the suitability areas reached the southernmost portion of the biome. The ENM models suggested the existence of multiple potential refuges for *Q. grandiflora*, with a large stable area in the central region of the Cerrado core and several other small stable areas at isolated peripheral sites.

## Discussion

### Genetic diversity, phylogeographic structure, and demographic history of Cerrado core

Our study on the most common and frequent tree species in the Cerrado, *Q. grandiflora*, indicated a phylogeographic structure shaped by climatic oscillations in the Pleistocene. A striking geographical structure observed was the separation of the cpDNA lineages belonging to the central-eastern cluster (CE) from the remaining ones, evidenced by the MJ network, BI analysis and, Monmonier's maximum difference algorithm. In the same way GENELAND analyses performed with cpDNA and nDNA data together reinforce this structure of the CE region. This separation between eastern and western Cerrado core was similarly observed for two other congeneric species, *Q. multiflora* and *Q. parviflora* (Buzatti et al., 2017). Additionally, this eastern-western split in the Cerrado was observed for several other plant species, such as Leguminosae tree species (Ramos et al., 2007, 2009; Novaes et al., 2013), an Asteraceae shrub (Collevatti et al., 2009), a Cactaceae species complex (Bonatelli et al., 2014), Annonaceae tree species (Ribeiro et al., 2016a,b) and a Malpighiaceae tree species (Resende-Moreira et al., 2017). This common pattern observed for phylogenetically distant species and with different habitus indicates a vicariant event in this region, probably related to the interplay between mountain ranges and climatic oscillations during the Quaternary.

The analysis of the historical colonization of *Q. grandiflora* lineages suggested that the mountain ranges of the Brazilian Shield substantially limited the colonization of eastern Cerrado (CE cluster). These mountain ranges are present from the northern until the southern portion of the putative barrier b estimated by the BARRIER analysis (Figure 3—left), and are known as Serra Geral plateau, Central Brazilian plateau, and Canastra range. This geographical feature could have enabled the first lineage colonization to eastern Cerrado, which occurred only from the south-western portion (cPAL population) at ca 720 ka (Figure 4C). Meanwhile, the presence of the mountain range appears to have limited posterior connections, demonstrated by the lineage colonization analysis, which showed that most of the colonization in the CE cluster occurred from the first-colonized area in warming periods (Figure 4F). Additionally, the founder effect associated to density-related processes and local adaptations could explain the low genetic diversity observed and maintenance of the west-east divergence pattern (Ribeiro et al., 2016a).

The central portion of the Cerrado core (CC cluster) exhibited high cpDNA diversity (Table 2). This diversity was consistent with previous studies and suggests the existence of a stable region, or refuge, wherein climatic variations in the Quaternary appear to have been less extreme (Ramos et al., 2007; Novaes et al., 2010, 2013; Ribeiro et al., 2016a; Buzatti et al., 2017; Souza et al., 2017). Our ENM analysis of *Q. grandiflora* and previous studies involving ENM predict that this central Cerrado region is located in a large climatically stable area, contributing significantly to the enhancement and maintenance of biodiversity (Terribile et al., 2012; Werneck et al., 2012; Collevatti et al., 2015; Bueno et al., 2017; Souza et al., 2017). In our study, the climatically stable area included within its south-western portion a population with high genetic diversity and exclusive haplotypes (cSER), which is a typical pattern of long-term large populations. High genetic diversity in the cSER area was similarly observed in a few studies wherein this region was sampled (Novaes et al., 2010—*Plathymenia reticulata*, Ribeiro et al., 2016a—*Annona coriacea*, and Buzatti et al., 2017—*Q. multiflora*). Moreover, small refuges in southern and western Cerrado were predicted highlighting that the diversity observed in this biome could have resulted from a complex scenario where cyclical isolation and recolonization processes from multiple refuges occurred. Besides, the high genetic diversity and genetic structure observed in *Q. grandiflora* can be a result from fast range contractions events with absence of gene flow between isolated areas (Arenas et al., 2012; Mona et al., 2014).

The putative ancestral population of *Q. grandiflora* was probably located at the north-western border of the CC cluster. During the Early Pleistocene (ca 1.49 Ma), the species began its colonization from this area toward the CC cluster and Amazonian savanna (aSAN population, see next subsection). Since this period, lineage colonization, mainly from the CC cluster, underwent a cyclical process with colonization toward the north occurring most in cooling periods. Contrastingly, in warming periods, lineages dispersed toward the eastern and southern portions of the Cerrado core, reaching latitudes beyond 20°S.

Southern Cerrado (SC cluster) showed low genetic diversity for cpDNA (Table 2), which was consistent with findings for other tree species and could be related to a severe restriction of species occurrence in southern Cerrado during Pleistocene glaciations (Collevatti et al., 2003; Ramos et al., 2007; Novaes et al., 2010, 2013; Buzatti et al., 2017; Souza et al., 2017). These results evidencing restriction in southern Cerrado during glacial periods are supported by palynological records, demonstrating that during cold and dry periods in the Quaternary vegetation at latitudes equal to or beyond 20°S was replaced by subtropical grasslands (Behling, 1998, 2002). Accordingly, our ENM results showed low suitability for *Q. grandiflora* at latitudes beyond 20°S in LGM.

Population cQGA, located within the Pantanal vegetation complex, showed only one exclusive haplotype (C27), phylogenetically close to the exclusive haplotype observed in a savanna enclave in south-western Amazonia (aHTA population). The RRW analysis showed that lineage colonization toward populations aHTA and cQGA from the central western began at approximately 1.17 Ma from the same region, corroborating the disjunction in clustering revealed by GENELAND. Population cPRI, located in a peripheral area in the transition between north-eastern Cerrado and Caatinga (a xeric vegetation type), did not show variation, displaying only a single haplotype (C04) shared with core populations. This agreed with the probable recent colonization of this area (0.45 Ma), demonstrated by the RRW analysis. In conclusion, our data for *Q. grandiflora* highlights the evolutionary complexity of ecotonal areas in the Cerrado, influenced by climatic changes in the Pleistocene.

### Relationships between Amazonian savannas and Cerrado core

The results obtained for *Q. grandiflora* did not support the replacement of a large continuous area of the Amazon forest by savanna vegetation, yet they did not fully refute Haffer's (1969) hypothesis of rainfall forest refuges in the Amazon forest. The high genetic divergence and putative barriers between the Amazonian savanna populations (aSAN and aHTA) suggested an ancient separation between these savanna enclaves of central and south-western Amazon forest. The Amazon Forest probably have restricted gene flow among Amazonian savanna populations, acting as an environmental barrier. Meanwhile, evidence of connections between the Cerrado core and Amazonian savannas was found, suggesting that the Cerrado expanded northwards, replacing a few areas in the Amazon forest during cold periods. Phylogeographic (Wüster et al., 2005; Quijada-Mascareñas et al., 2007), species distribution (Webb, 1991; Avila-Pires, 1995; Silva, 1995) and ENM studies (Werneck et al., 2012; Bueno et al., 2017) suggest connections between the Amazonian savanna and Cerrado core; however, there is no consensus about where and when they occurred.

The *Q. grandiflora* population in the savanna enclave in central Amazonia (aSAN) constituted an isolated cluster, wherein only one endemic and divergent (i.e., with at least seven mutational steps) haplotype was observed, evidencing an ancient isolation of the population aSAN from the Cerrado core. Furthermore, the RRW model showed that lineages probably dispersed to aSAN only by a central corridor at the end of a cold period in the Early Pleistocene (ca 1.49 Ma). Later, this region was isolated by a vicariant process, probably by Amazon forest expansion. The nDNA evidenced similar findings, with most of the haplotypes exclusive to the population aSAN. However, for this genetic nuclear marker, two haplotypes were shared with populations located in the central portion of the Cerrado core and one of them was shared with cPRI as well. These data suggested the existence of a central connection between the core and aSAN (known as the Central corridor or “Amazonian Dry Corridor”) yet did not exclude a putative connection between aSAN and cPRI through Coastal corridor. However, cPRI and aSAN could also have independently received the same nDNA haplotype from the core. The ENM studies on *Q. grandiflora* predicted the suitability area at the same place where the Central corridor could have occurred during LIG. Furthermore, a recent study based on uranium/thorium dating and oxygen isotopic records obtained for stalagmites in a cave located near the population aSAN (Wang et al., 2017) suggests the existence of a dry corridor comprising dry forest habitats, not savannas, during the most recent ice age from 24 to 18 ka. However, neither our genetic findings nor the ENM prediction contradict the study by Wang et al. (2017), since they indicated the existence of a more ancient connection between the Cerrado core and central Amazon forest.

Apart from the ancient connection between the Cerrado core and central Amazonian savanna, evidence of an ancient connection was found between the Cerrado core and another savanna enclave in south-western Amazon (aHTA). The *Q. grandiflora* population in this enclave possessed only an exclusive cpDNA haplotype, closer to the only haplotype present in population cQGA and the most common haplotype observed in the populations in southern Cerrado, indicating the existence of another connection with the Cerrado core. Lineages dispersed from central Cerrado to western Cerrado at ca 1.09 Ma and later (ca 830 ka, a cold period in the Early Pleistocene), from this area, colonized cQGA and a small putative refuge near aVHA and aHTA, as predicted by the ENM analysis. Later, in the Middle Pleistocene, this refuge appeared to be the source of lineages constituting populations aVHA and aHTA, which could explain the relative genetic proximity between cQGA and aHTA. Populations aHTA and aVHA were clustered together in GENELAND analysis, probably due they common ancestry. In the same way, cQGA population showed high probability of belonging both to the same cluster as aHTA and aVHA or cSER. These results together corroborate the species colonization routes inferred by RRW analysis and also could explain the pattern obtained in the GENELAND non-spatialized model (Supplementary Figure 3) in which these four populations (aHTA, aVHA, cQGA and cSER) were grouped together. The single exclusive cpDNA lineage observed in aVHA was phylogenetically close to one lineage observed only in the cPAL population (central Cerrado). Altogether, these results suggested an expansion from central Cerrado toward the north-western portion of the biome, corroborating the results obtained by Buzatti et al. (2017) for *Q. multiflora* and *Q. parviflora*.

The genetic divergence between south-western and central Amazonian savannas observed for cpDNA was similarly observed for nDNA. The haplotypes obtained in aHTA were exclusive and genetically distant from the nDNA haplotypes of aSAN (central Amazonian savanna) and aVHA (located at western Cerrado border at the Cerrado/Amazon forest transition). The different connections observed between the Cerrado core and central and south-western Amazonian savannas in different cooling periods could be related to a dichotomous pattern of response to climatic changes between the western (aHTA side) and eastern (aSAN side) Amazonian portions described by Cheng et al. (2013). According to them, speleothem δ^18^O records indicate that western portion Amazon forest remained wetter than the eastern side, reinforcing the non-connection between populations aHTA and aSAN.

This is the first time a genetic study on plant species in the Cerrado evidenced a central connection between the Cerrado core and Amazonian savannas. According to our results, these connections occurred during a remote period, before LIG or LGM. The Amazonian savannas appear to be fragmented and isolated from each other, evolving independently a long ago. Our study brings insights into the historical relationships between the Cerrado core and Amazonian savannas. However, due to intrinsic stochasticity of the coalescent processes and influence of other features such as life histories traits, more studies with increased marker numbers, more Amazonian savanna samples and analysis of other species are necessary to improve our knowledge about the evolutionary history of the Amazonian savannas.

Our findings reinforce that the central part of Cerrado core is an important area for the conservation of the biome genetic diversity. Additionally, other small stable areas during Pleistocene climatic oscillations with exclusive haplotypes and/or high diversity are important to ensure the evolutionary potential of the species under predicted future climatic changes. Thus, these multiple potential refuges should be included in conservation programs. Special attention should be paid to poorly known Amazonian savannas, which show exclusive haplotypes and independent evolution of Cerrado core.

## Author contributions

RB, JL-F, ML planned and designed the research. RB performed laboratory work. RB, ML, and JL-F collected the population samples. ML and JL-F provide financial resources to work. RB, TP, MB and RM carry out the data analyzes. All authors contributed to the manuscript writing, mainly RB and ML.

### Conflict of interest statement

The authors declare that the research was conducted in the absence of any commercial or financial relationships that could be construed as a potential conflict of interest.
