# Supplementary material for: Genetic and Historical Colonization Analyses of an Endemic Savanna Tree, Qualea grandiflora, Reveal Ancient Connections Between Amazonian Savannas and Cerrado Core
Source: Front Plant Sci. 2018 Jul 17;9:981. doi: 10.3389/fpls.2018.00981 (PMC6056688; doi:10.3389/fpls.2018.00981)
Supplement: Supplementary file 1 [file Data_Sheet_1.doc]

Supplementary Material

**Genetic and Historical Colonization Analyses of an Endemic Savanna Tree, *Qualea grandiflora*, Reveal Ancient Connections Between Amazonian Savannas and Cerrado Core**

**Renata Santiago de Oliveira Buzatti^1^, Thais Ribeiro Pfeilsticker^1^, Rafael Félix de Magalhães^3^, Marcelo Leandro Bueno^3^, José Pires Lemos-Filho^4^, Maria Bernadete Lovato^1^**^*^

*** Correspondence:** lovatomb@icb.ufmg.br

# Supplementary Data

**Supplementary Material and Methods**

The primers used to amplify the single copy gene AGT1 were designed by our research group using sequences obtained from GenBank via Basic Local Alignment Search Tool (P. Vasconcelos, UFMG, Belo Horizonte, Brazil, unpubl.res.). The polymerase chain reaction (PCR) was performed in a 25µl total volume, for all regions, under the following conditions: 10 - 20 ng of genomic DNA, 1X PCR buffer [2.0 mM of MgCl_2_, 10 mM Tris–HCl (pH 8.4), 50 mM of KCl, 0.1% Triton X-100 (Phoneutria, Belo Horizonte, Brazil)], 200 mM of each dNTP, 0.2 ng of bovine serum albumin (BSA), 0.5mM of each primer, 1 U Taq DNA polymerase (Phoneutria, Belo Horizonte, Brazil), and autoclaved deionized water. Amplification conditions for cpDNA regions were constituted by initial denaturation at 94°C for 4 min, followed by 35 cycles at 94ºC for 1 min, 54°C (*trn*A*-trn*H), 56°C (*trn*S*-trn*G) for 1 min, and 72°C for 1 min, and a final elongation at 72°C for 7 min. For nDNA, the amplification program consisted of an initial denaturation at 96°C for 2 min, followed by 35 cycles at 94ºC for 45 seg, 51°C for 1 min, and 72°C for 1 min and 30 seg, and a final elongation at 72°C for 10 min. Successful amplification was checked on a 1% agarose gel and later purified with 20% polyethylene glycol. Then, the purified PCR products generated from *trn*S*-trn*G were double-strand sequenced using the DYEnamic ET dye terminator sequencing Kit (GE Healthcare, UK) in a MegaBACE 1000 automated sequencer (GE Healthcare). The purified PCR products generated from *trn*A*-trn*H and AGT1 were double-strand sequenced using the ABI Big Dye v.3.1 terminators in an ABI3130XL automated sequencer (Applied Biosystems, Foster City, CA).

# Supplementary Figures and Tables

## Supplementary Figures

**Supplementary Figure 1.** Natural geographic distribution of *Qualea grandiflora*. Data from NeoTropTree (red dots, Oliveira-Filho, 2014) and our samples (green dots)


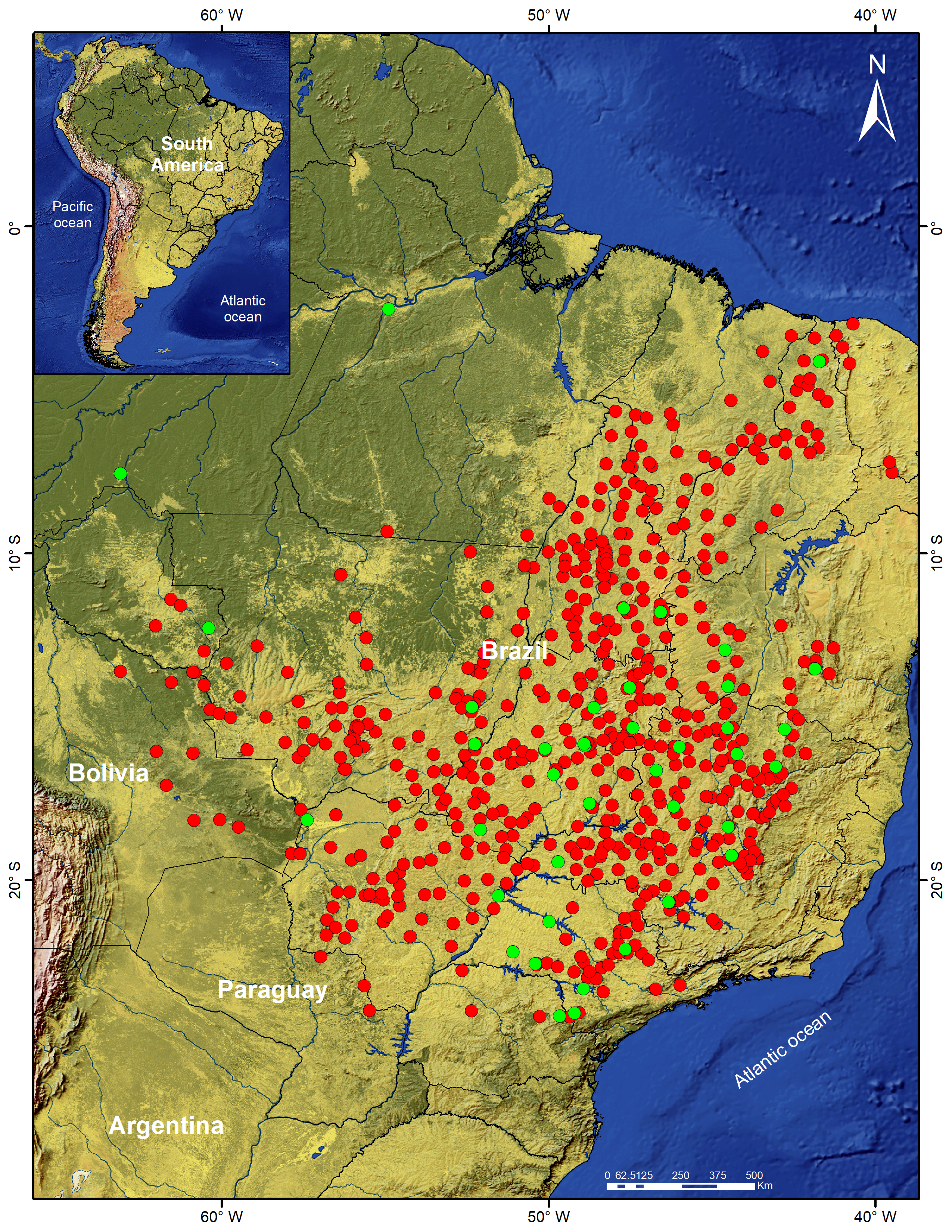


**Supplementary Figure 2.** Traces of posterior probability of the EBSP model along states of the Markov chain Monte Carlo (MCMC) for first run (A), second run (B) and combined runs (C), showing stationarity (A and B) and convergence (C). Gray traces (A and B) refer to discarded samples (i.e., burn-in). No parameters show ESS < 300 in the combined results.


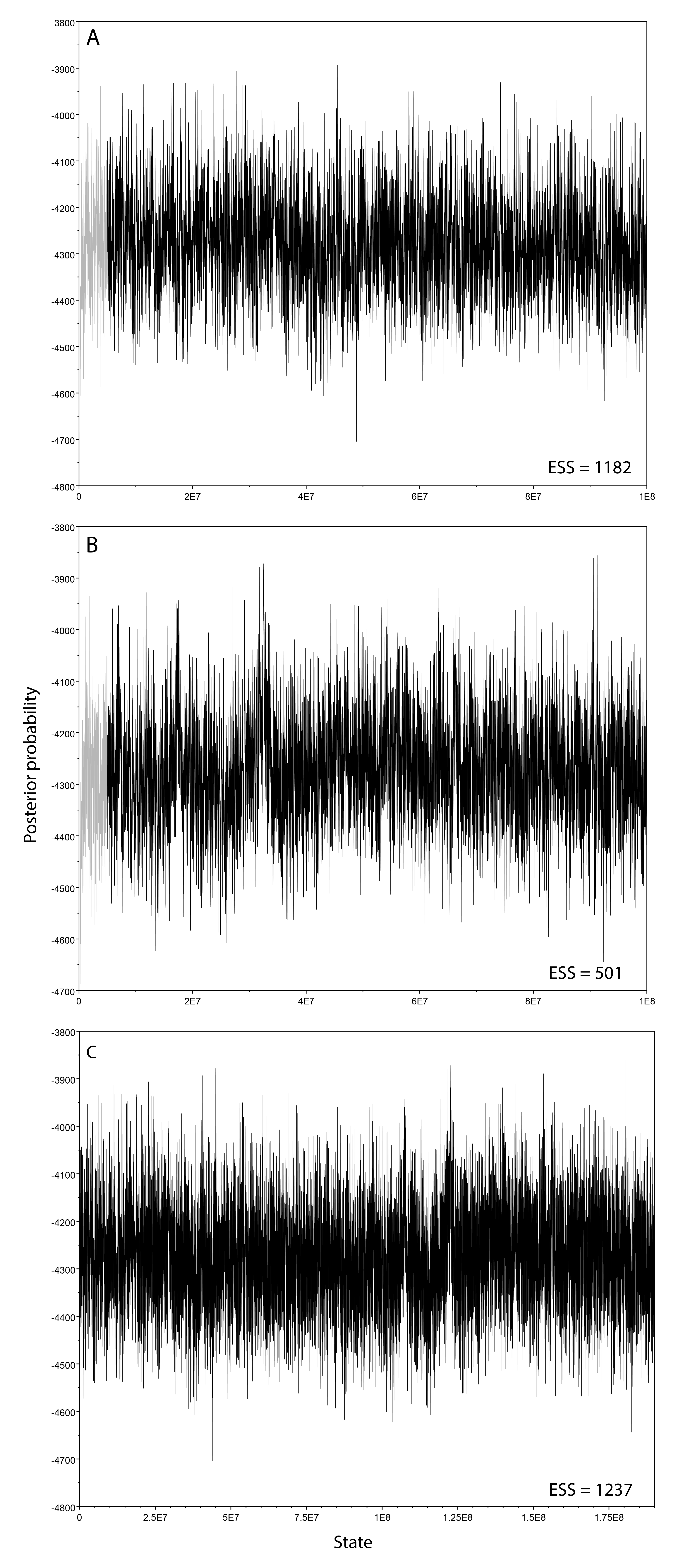


**Supplementary Figure 3.** Non-spatialized population structure of *Qualea grandiflora* determined by Bayesian analysis using GENELAND based on nDNA and concatenated cpDNA sequences. Barplot shows the density in relation to the number of clusters through MCMC.


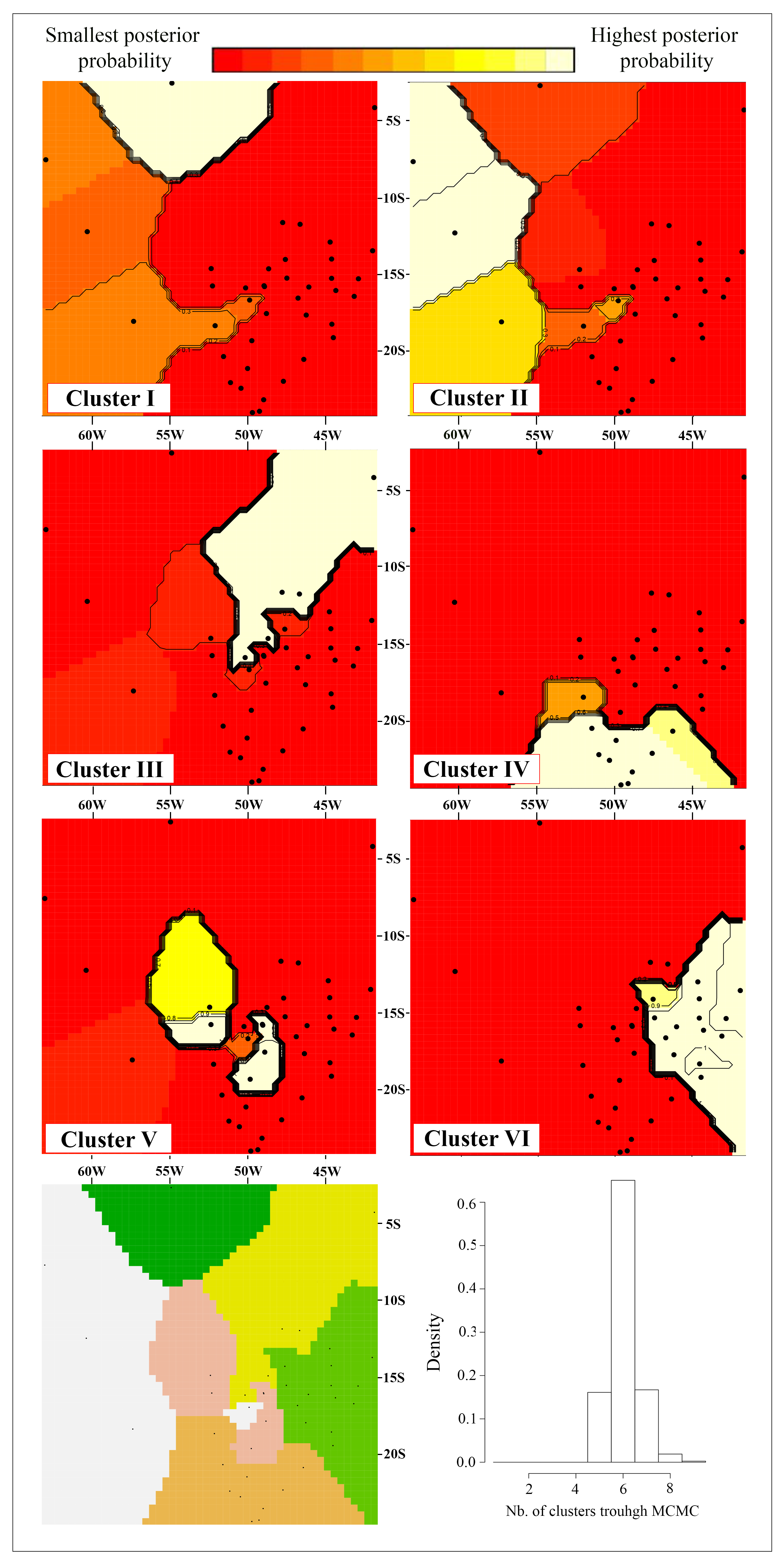


**Supplementary Figure 4.** Bayesian phylogenetic tree showing the evolutionary relationship of *Qualea grandiflora* based on concatenated cpDNA region. Posterior probabilities higher than 0.90 are shown above the branches. Branch colors are referents to the lineages showed in the MJ network (main text)


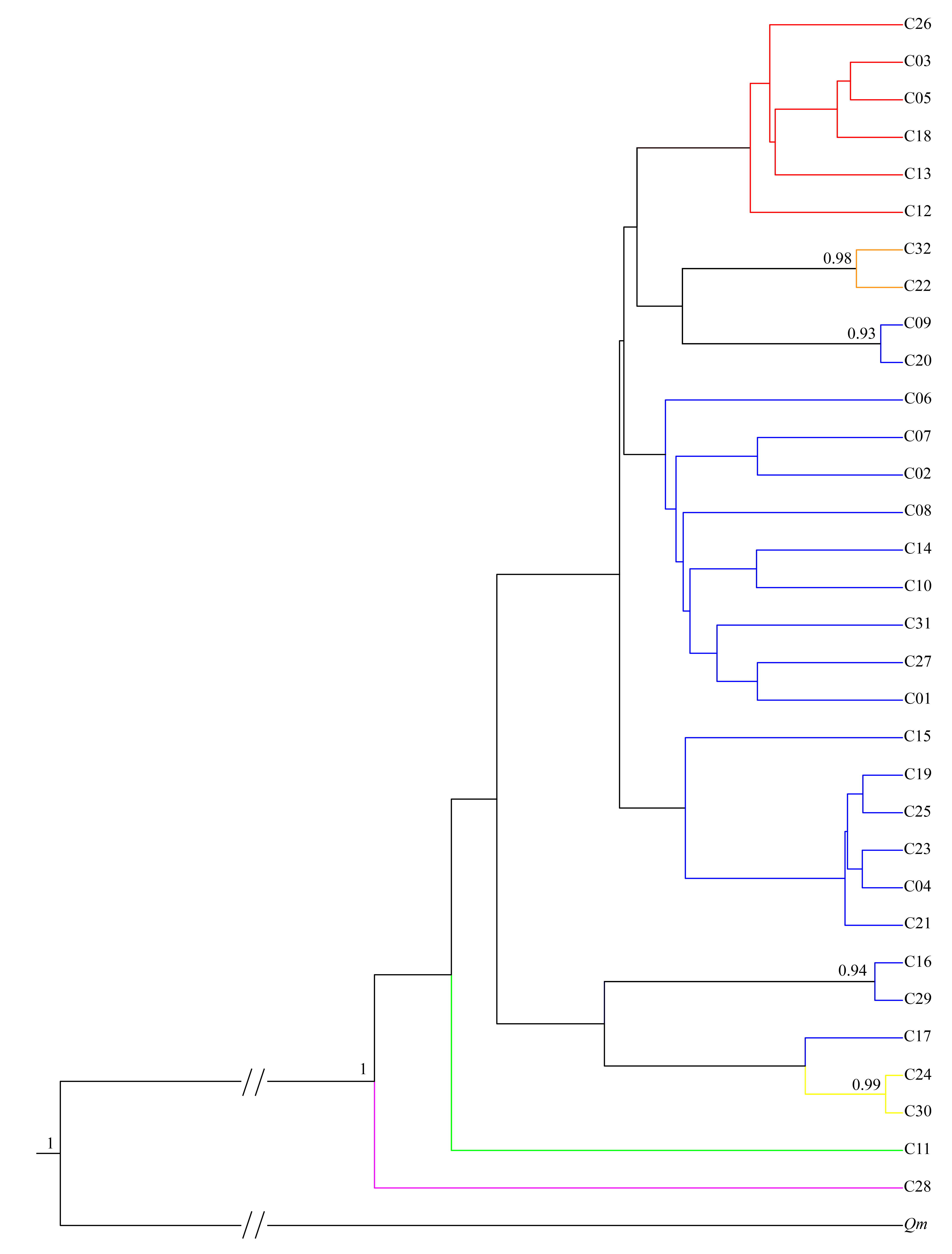


**Supplementary Figure 5.** Bayesian phylogenetic tree showing the evolutionary relationship of *Qualea grandiflora* based on nDNA region. Posterior probabilities higher than 0.90 are shown above the branches. Branch colors are referents to the lineages showed in the MJ network (main text)


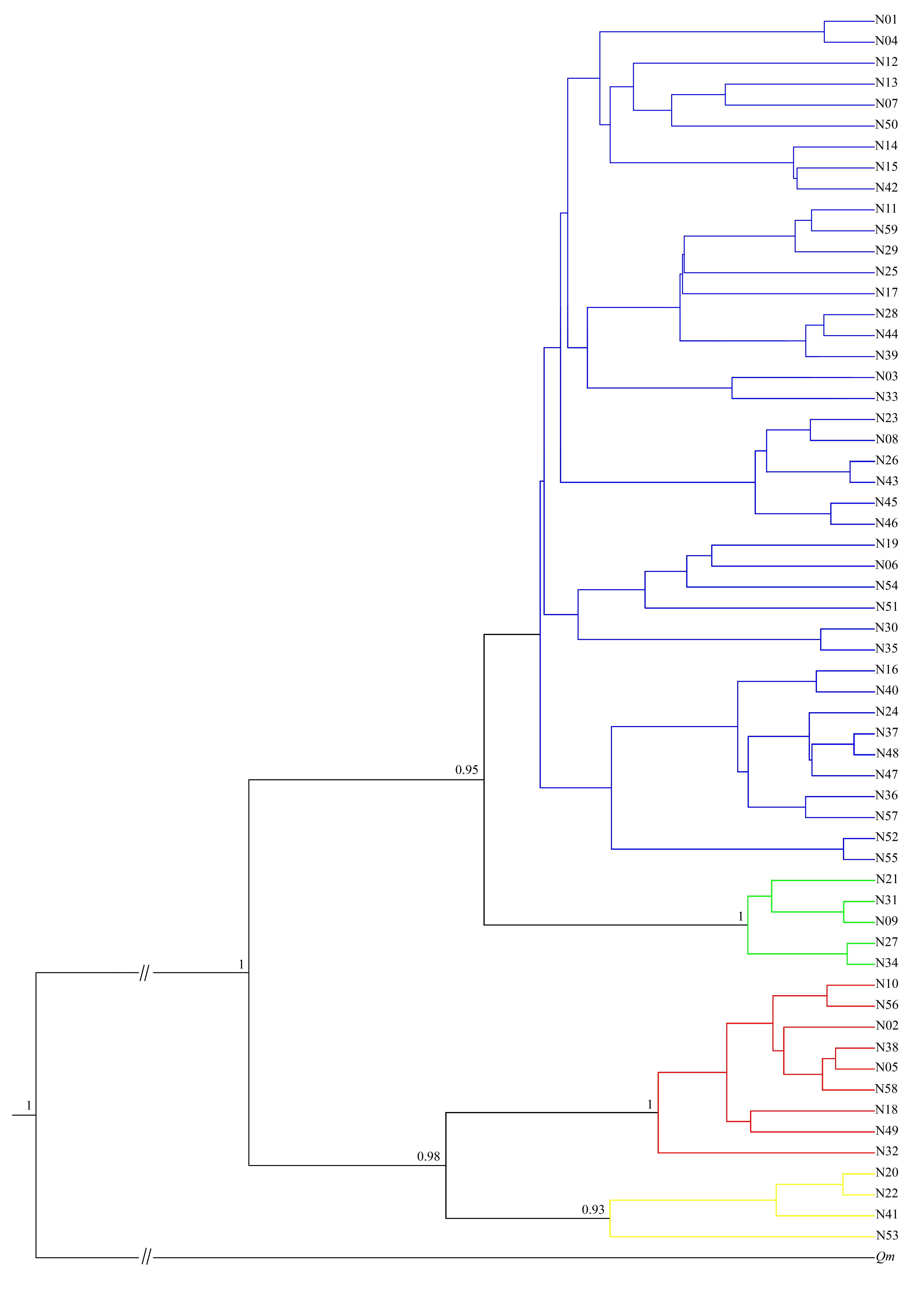


**Supplementary Figure 6.** Population size over time of *Qualea grandiflora* estimated with Extended Bayesian Skyline Plot, where the dashed line is the median and the gray area is the credible posterior density of the relative effective population size (A). Histogram of tree event times in logarithm, showing the high density of population changes in the very recent period (B). The photographs illustrate, from top to bottom, a ripe fruit, flowers and an adult individual of the species.


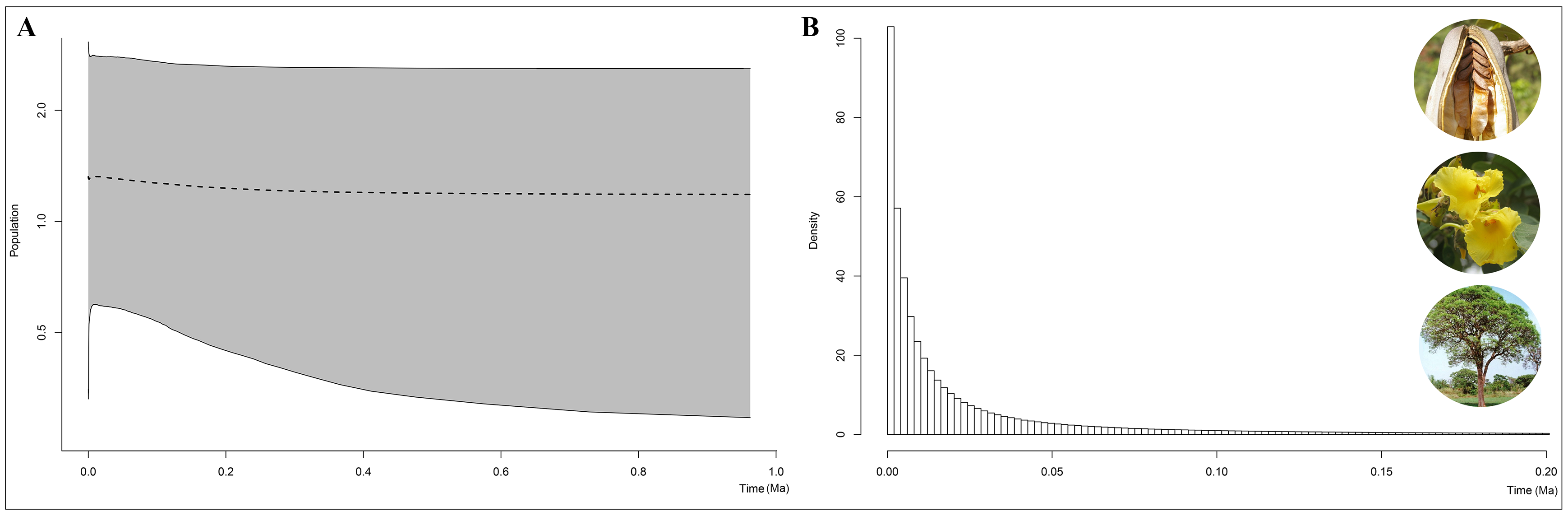


**Supplementary Figure 7.** Bayesian phylogenetic tree showing the evolutionary relationship of *Qualea grandiflora* based on concatenated cpDNA region, obtained from Relaxed Random Walk analysis. Nodes with posterior probabilities higher than 0.50 are highlighted with a black dot. Nodes with posterior probabilities higher than 0.90 are highlighted with a square and the probability values are shown above the branches.





## Supplementary Tables

**Supplementary Table 1.** List of populations used in this study with herbarium records.

**Supplementary Table 2. Primer sequences and reference of the 30 primer pairs used for screening the markers with good amplification and polymorphism of chloroplast DNA in *Qualea grandiflora*.**

|  |  |  |
| --- | --- | --- |
|  | cpDNA region | Reference |
| 1 | MatK | Sang et al., 1997 |
| 2 | trnQ-rps16 | Shaw et al. 2007 |
| 3 | trnS-trnG | Hamilton, 1999 |
| 4 | trnH-psbA | Sang et al., 1997 |
| 5 | trnV-trnM | Demesure et al. 1995 |
| 6 | psbC-trnS3 | Demesure et al. 1995 |
| 7 | rpl32-trnL | Shaw et al. 2007 |
| 8 | trnD-trnT | Shaw et al. 2005 |
| 9 | rps16-trnK | Shaw et al. 2005 |
| 10 | psaA-trnS | Demesure et al. 1995 |
| 11 | atpB-rbcL | Hodges and Arnold 1994 |
| 12 | trnlC-trnlF | Taberlet et al. 1991 |
| 13 | psbD-trnT | Shaw et al. 2007 |
| 14 | trnH-trnK | Demesure et al. 1995 |
| 15 | psbJ-petA | Shaw et al. 2007 |
| 16 | petL-psbE | Shaw et al. 2007 |
| 17 | ndhF-rpl32 | Shaw et al. 2007 |
| 18 | rpoB-trnC | Shaw et al. 2005 |
| 19 | rpL20-rpS12 | Shaw et al. 2005 |
| 20 | atpI-atpH | Shaw et al. 2007 |
| 21 | ndhaX1-ndhaX2 | Shaw et al. 2007 |
| 22 | trnV-ndhC | Shaw et al. 2007 |
| 23 | trnK1-trnK2 | Demesure et al. 1995 |
| 24 | ccmp4L-atpH | Shaw et al. 2007 |
| 25 | psbB-psbF | Hamilton, 1999 |
| 26 | trnQ-trnS2 | Shaw et al. 2007 |
| 27 | accD-psai75R | Small et al. 1998 |
| 28 | rpl14-rpl36 | Shaw et al. 2007 |
| 29 | ndhJ-tabE | Shaw et al. 2007 |
| 30 | trnT-trnL | Taberlet et al. 1991 |

**Supplementary Table 3.** GenBank accession number for the two cpDNA regions (A) and nuclear gene (B) used in this study.

**A**

**B**

**Supplementary Table 4.** Evaluation of the model performance for *Qualea grandiflora* by Maxent, True skill statistic (TSS), and area under the curve (AUC).

**References**

Hamilton MB (1999) Four primer pairs for the amplification of chloroplast intergenic regions with intraspecific variation. *Mol. Ecol.* 8:521–523.

Hodges, S. A., and Arnold M.L. (1994) Columbines: a geographically widespread species flock. *Proc. Natl. Acad. Sci.*, USA 91: 5129-5132.

Shaw J, Lickey E, Beck JT, Farmer SB, Liu W, Miller J, Siripun KC, Winder CT, Schilling EE, Small RL. (2005) The tortoise and the hare II: relative utility of 21 noncoding chloroplast DNA sequences for phylogenetic analysis. *Am. J. Bot.* 92: 142–166.

Shaw J, Lickey EB, Schilling EE, Small RL. (2007) Comparison of whole chloroplast genome sequences to choose noncoding regions for phylogenetic studies in angiosperms: the tortoise and the hare III. *Am. J. Bot.* 94: 275–288.

Small R. L., Ryburn J. A., Cronn R. C., Seelanan T. and Jonathan F. Wendel J. F. (1998). The Tortoise and the Hare: Choosing between Noncoding Plastome and Nuclear Adh Sequences for Phylogeny Reconstruction in a Recently Diverged Plant Group. *Am. J. Bot.* 85, 1301-1315.

Taberlet P, Gielly L, Pautou G, Bouvet J (1991) Universal primers for amplification of three non-coding regions of chloroplast DNA. *Plant. Mol. Biol.* 17:1105–1109
